# Real-Time Imaging Reveals Augmentation of Glutamate-Induced Ca^2+^ Transients by the NO-cGMP Pathway in Cerebellar Granule Neurons

**DOI:** 10.3390/ijms19082185

**Published:** 2018-07-26

**Authors:** Michael Paolillo, Stefanie Peters, Andrea Schramm, Jens Schlossmann, Robert Feil

**Affiliations:** 1 Interfaculty Institute of Biochemistry, University of Tübingen, 72076 Tübingen, Germany; michael.paolillo@uni-tuebingen.de (M.P.); stefanie.peters07@gmail.com (S.P.); 2 Graduate School of Cellular and Molecular Neuroscience, University of Tübingen, 72074 Tübingen, Germany; 3 Department of Pharmacology and Toxicology, University of Regensburg, 93053 Regensburg, Germany; andrea.schramm@chemie.uni-regensburg.de (A.S.); jens.schlossmann@chemie.uni-regensburg.de (J.S.)

**Keywords:** cyclic GMP, calcium, nitric oxide, guanylyl cyclase, cerebellar granule cells, protein kinase, PKG, FRET imaging, transgenic mice

## Abstract

Dysfunctions of NO-cGMP signaling have been implicated in various neurological disorders. We have studied the potential crosstalk of cGMP and Ca^2+^ signaling in cerebellar granule neurons (CGNs) by simultaneous real-time imaging of these second messengers in living cells. The NO donor DEA/NO evoked cGMP signals in the granule cell layer of acute cerebellar slices from transgenic mice expressing a cGMP sensor protein. cGMP and Ca^2+^ dynamics were visualized in individual CGNs in primary cultures prepared from 7-day-old cGMP sensor mice. DEA/NO increased the intracellular cGMP concentration and augmented glutamate-induced Ca^2+^ transients. These effects of DEA/NO were absent in CGNs isolated from knockout mice lacking NO-sensitive guanylyl cyclase. Furthermore, application of the cGMP analogues 8-Br-cGMP and 8-pCPT-cGMP, which activate cGMP effector proteins such as cyclic nucleotide-gated cation channels and cGMP-dependent protein kinases (cGKs), also potentiated glutamate-induced Ca^2+^ transients. Western blot analysis failed to detect cGK type I or II in our primary CGNs. The addition of phosphodiesterase (PDE) inhibitors during cGMP imaging showed that CGNs degrade cGMP mainly via Zaprinast-sensitive PDEs, most likely PDE5 and/or PDE10, but not via PDE1, 2, or 3. In sum, these data delineate a cGK-independent NO-cGMP signaling cascade that increases glutamate-induced Ca^2+^ signaling in CGNs. This cGMP–Ca^2+^ crosstalk likely affects neurotransmitter-stimulated functions of CGNs.

## 1. Introduction

NO acts as an important messenger in the nervous system. It elevates the intracellular concentration of cGMP, which activates downstream targets and ultimately leads to a variety of neurophysiological responses [1]. The NO-cGMP pathway has been implicated in several aspects of the central nervous system (CNS), such as cognition, anxiety, addiction, schizophrenia, and depression [2]. NO increases cGMP levels via activation of the hemeprotein NO-sensitive guanylyl cyclase (NO-GC), which subsequently synthesizes cGMP from GTP. NO-GC is a heterodimeric enzyme thought to be mainly localized in the cytosol. It exists in two isoforms, α_1_β_1_ and α_2_β_1_, where α_1_β_1_ is more prevalent in most tissues, except for neural tissue where comparatively higher levels of α_2_β_1_ are found [3,4,5]. cGMP synthesis is also stimulated via activation of transmembrane particulate guanylyl cyclases [6]. Activation of these enzymes occurs via peptides such as atrial natriuretic peptide (ANP) or C-type natriuretic peptide (CNP).

cGMP exerts its physiological effects via the modulation of at least three types of downstream effectors: cGMP-dependent protein kinases (cGKs), cyclic nucleotide-gated (CNG) cation channels, and cGMP-regulated phosphodiesterases (PDEs). Mammals express three cGKs, membrane bound cGKII, and cytosolic cGKI, which has two isoforms, known as cGKIα and cGKIβ [7]. Both cGKI and cGKII are expressed in the CNS [8,9,10]. CNG channels have also been identified in the nervous system [11,12]. They consist of at least two types of subunits, termed α and β, and exist in at least five isoforms. CNG channels are directly activated by cGMP and/or cAMP and provide a non-voltage dependent route for Ca^2+^ entry into neurons, in addition to voltage-activated Ca^2+^ channels and plasma membrane channels gated by excitatory neurotransmitters such as glutamate and acetylcholine. cGMP is degraded by PDEs [13]. Interestingly, cGMP also regulates the activity of some cAMP-hydrolyzing PDEs, thus, facilitating cross regulation of cAMP levels by cGMP. For example, binding of cGMP to PDE2 increases the hydrolytic activity of the enzyme, resulting in a reduction in the cAMP concentration [14,15].

cGMP is implicated in various diseases, and multiple medications that increase the cGMP concentration are already on the market for use in humans, such as the PDE5 inhibitor Sildenafil for erectile dysfunction, the guanylyl cyclase C agonist Linaclotide for chronic idiopathic constipation and irritable bowel syndrome, and the NO-GC stimulator Riociguat for certain forms of pulmonary hypertension [16,17,18,19]. Given the druggability of the cGMP pathway, it would be informative to further elucidate the cGMP signaling pathway in the nervous system. cGMP has important functions in various regions of the brain including the cerebellum [2]. A NO-cGMP-cGKI pathway regulates long-term-depression of synaptic activity in Purkinje cells and cerebellar learning [20]. Expression of components of the NO-cGMP pathway has also been reported in cerebellar granule neurons (CGNs) [21], but the functional relevance of NO-cGMP signaling in the granule cell layer of the cerebellum is not well understood. CGNs are glutamatergic interneurons that provide an excitatory input to the molecular layer of the cerebellum. They constitute the largest neuronal population in the cerebellum and entire brain and serve as a model to investigate neuronal signal transduction [22]. CGNs have been shown to express Ca^2+^-permeable *N*-methyl-d-aspartate (NMDA) receptors for glutamate [23,24]. These receptors are essential for neuronal survival and previous research has reported that upon NMDA receptor stimulation, NO synthase is activated and NO is synthesized [25,26].

**Figure 1:**
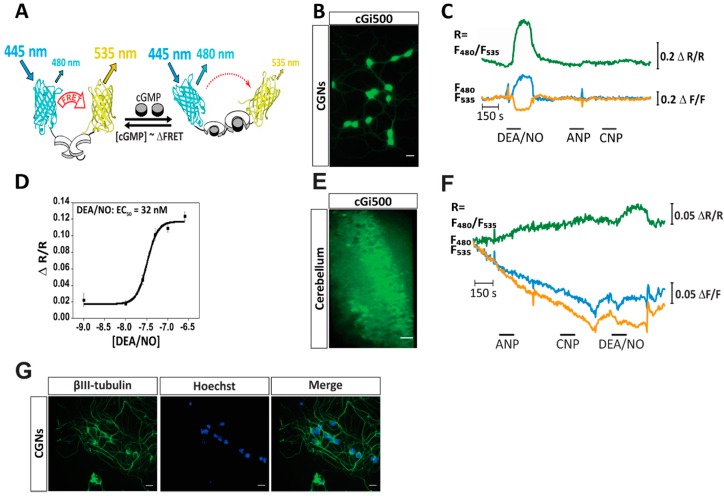
Visualization of cGMP in CGNs and acute cerebellar slices revealed a NO-induced cGMP response. (**A**) The cGi500 sensor consisted of cGMP-binding sites of the bovine cGKI (white) flanked by CFP (cyan) and YFP (yellow). Upon binding of cGMP (grey) a conformational change and a concomitant decrease in FRET efficiency occurred. Thus, upon excitation of CFP at 445 nm, light emission from YFP (535 nm) was reduced and emission from CFP (480 nm) was increased resulting in an increase of the CFP/YFP emission ratio when cGMP concentrations rise. Adapted from Reference [27]. (**B**) CGNs isolated from 7-day-old R26-CAG-cGi500(L1) mice expressed the cGi500 sensor (green). The green color represents the YFP fluorescence of cGi500. Scale bar 10 μm. (**C**) cGMP measurement in CGNs upon stimulation with DEA/NO (100 nM), ANP (100 nM), and CNP (100 nM). Green, cyan, and orange traces represent CFP/YFP ratio, CFP emission, and YFP emission, respectively. Scale bars represent the change in fluorescence of the single fluorophores CFP or YFP (ΔF/F) or their ratio (CFP/YFP; ΔR/R reflecting [cGMP]). The signals are from a single cell representative of the data set (*n* = 57 cells measured on six coverslips obtained from three independent cultures). (**D**) Increasing concentrations of DEA/NO were applied to CGNs to establish a concentration-response curve. The EC_50_ of CGNs for DEA/NO was ≈32 nM. Data are presented as mean ± SEM (*n* = 12 cells per data point; cells from one culture were measured on one coverslip). (**E**) Acute cerebellar slice isolated from a 7-week-old R26-CAG-cGi500(L1) mouse. The green color represents the YFP fluorescence of cGi500. Scale bar is 20 µm. (**F**) cGMP measurement in the granule cell layer of a cerebellar slice upon stimulation with ANP (250 nM), CNP (250 nM), and DEA/NO (5 µM). Green, cyan, and orange traces represent CFP/YFP ratio, CFP emission, and YFP emission, respectively. Scale bars represent the change in fluorescence of the single fluorophores CFP or YFP (ΔF/F) or their ratio (CFP/YFP; ΔR/R reflecting [cGMP]). The signals are from a single region of interest representative of the data set (*n* = 3 acute cerebellar slices analyzed in three independent experiments). (**G**) Representative picture of a CGN primary culture stained for β_III_-tubulin (neuronal marker, green) and nuclei with Hoechst dye (blue); an overlay of both channels is also shown (Merge). Scale bar is 10 µm.

To investigate the NO-cGMP pathway in CGNs, in particular potential cGMP–Ca^2+^ crosstalk, we established primary cultures of CGNs from cGMP sensor mice and performed fluorescence imaging experiments to visualize both NO-induced cGMP and glutamate-induced Ca^2+^ in real time. cGMP was monitored via the fluorescence resonance energy transfer (FRET)-based cGMP indicator, cGi500, that was inserted as a transgene into the mouse genome [27]. As illustrated in Figure 1A, cGi500 consists of the two cGMP-binding sites of the bovine cGKI flanked by cyan fluorescent protein (CFP) and yellow fluorescent protein (YFP) [28]. It is a ratiometric sensor and indicates an increase in cGMP via reduced FRET. Ca^2+^ imaging was performed simultaneously with the ratiometric Ca^2+^-sensitive fluorescent dye Fura-2. By simultaneous cGMP/Ca^2+^ imaging, this study has identified a NO-cGMP signaling cascade that generates cGMP–Ca^2+^ crosstalk in murine CGNs that might affect neurotransmitter-dependent functions in the cerebellum.

## 2. Results

### 2.1. cGMP Imaging in CGNs and Acute Cerebellar Slices Reveals a NO-Induced cGMP Response

Primary CGN cultures were prepared from 7-day-old R26-CAG-cGi500(L1) mice [27], which express the cGi500 sensor ubiquitously. As expected, CGNs showed a strong expression of the cGMP sensor protein (Figure 1B, green fluorescence). It is important to note that the green fluorescence shown in Figure 1B shows the YFP emission of the sensor and not the cGMP concentration, which is reflected by the CFP/YFP emission ratio. To determine which substances can induce a reliable elevation of cGMP in CGNs, the cells were exposed to ANP (100 nM), CNP (100 nM), or the NO-releasing compound diethylamine NONOate (DEA/NO) (100 nM) during real-time imaging under continuous flow. As documented in Figure 1C, application of DEA/NO induced a robust cGMP elevation as indicated by a simultaneous increase in CFP emission (cyan trace) and decrease in YFP emission (orange trace), resulting in an increased CFP/YFP ratio (green trace). Application of ANP or CNP did not result in a cGMP increase. To determine the sensitivity of the cGMP response of CGNs to DEA/NO, a concentration-response experiment was performed, revealing an EC_50_ of ≈32 nM (Figure 1D). cGMP was also visualized in freshly prepared cerebellar slices isolated from R26-CAG-cGi500(L1) mice (Figure 1E). Cerebellar slices were exposed to ANP (250 nM), CNP (250 nM), and DEA/NO (5 µM) during real-time imaging under continuous flow. As expected, the application of DEA/NO, but not ANP or CNP, resulted in elevated cGMP in the granule cell layer of the cerebellum (Figure 1F, green trace). The failure to detect ANP- or CNP-induced cGMP increases (Figure 1C,F) could have been related to technical issues, such as an inability of the cytosolic cGi500 sensor to detect presumably membrane-associated cGMP pools that are generated in response to natriuretic peptides. However, this possibility is highly unlikely, because the cytosolic cGi500 sensor has been successfully used to detect ANP- and CNP-induced cGMP signals in various other cell types including smooth muscle cells [27] and dorsal root ganglion neurons [29].

To confirm that our cultured cells were indeed neurons, cells were stained for the neuronal marker β_III_-tubulin (Figure 1G). Over 95% of cells (detected by nuclear staining with Hoechst dye) were positive for β_III_-tubulin indicating a nearly pure neuronal cell culture. The cultured cells had the typical morphology and small size (diameter ≈ 5–10 µm) of CGNs (Figure 1B), but we cannot formally exclude that our cerebellar cultures also contained a small number of Golgi and/or stellate neurons. Together, these data indicated that NO was able to reliably elevate cGMP in CGNs in the primary culture as well as in acute cerebellar slices.

### 2.2. Simultaneous Imaging Implicates the NO-cGMP Pathway in Potentiation of Glutamate-Induced Ca^2+^ Transients in CGNs

**Figure 2:**
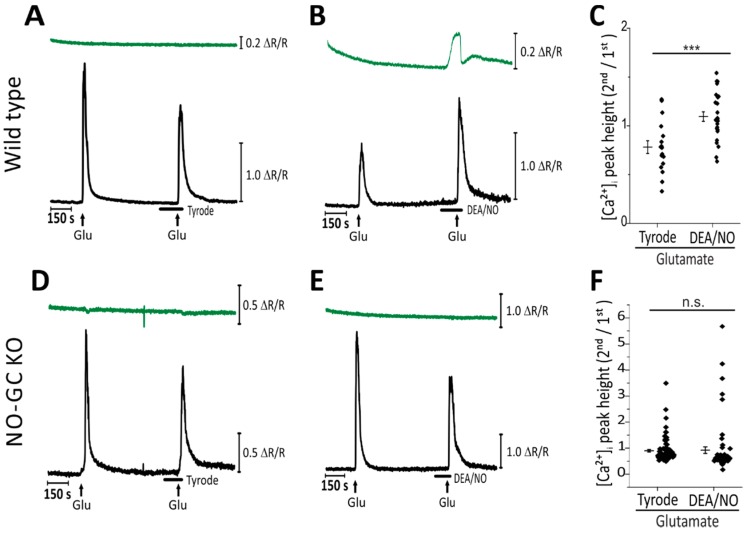
Simultaneous measurement of cGMP and Ca^2+^ revealed cGMP–Ca^2+^ crosstalk in CGNs. Traces depict cGMP (green) visualized with the cGi500 sensor, and [Ca^2+^]_i_ (black) visualized with Fura-2. CGNs were exposed to glutamate (100 μM) (for 10 s, represented by vertical arrows) to elevate [Ca^2+^]_i_, and to DEA/NO (100 nM) (represented by horizontal bars) to increase the cGMP concentration. Tyrode buffer was used as a control in the absence of DEA/NO. Traces depict representative measurements of individual cells. Scale bars represent the change in fluorophore ratio (ΔR/R reflecting [cGMP] or [Ca^2+^]_i_). (**A**–**C**) Imaging of cGi500-expressing CGNs with NO-GC wild type alleles (wild type). (**A**) Brief repetitive stimulation with glutamate under control conditions. (**B**) CGNs were exposed to glutamate alone and then to DEA/NO, followed by a second stimulation with glutamate in the presence of DEA/NO. (**C**) Quantification of the results obtained under conditions shown in (**A**,**B**). Depicted is the ratio of the [Ca^2+^]_i_ peak heights (second over first peak) of individual cells. Data are presented as mean ± SEM; *** *p* < 0.001 (*n* = 17 cells for Tyrode, *n* = 25 cells for DEA/NO, measured on six coverslips obtained from three independent cultures). (**D**–**F**) Imaging of cGi500-expressing CGNs with a genetic deletion of NO-GC (NO-GC KO). (**D**) Brief repetitive stimulation with glutamate under control conditions. (**E**) CGNs were exposed to glutamate alone and then to DEA/NO followed by a second stimulation with glutamate in the presence of DEA/NO. (**F**) Quantification of the results obtained under conditions shown in (**D**,**E**). Depicted is the ratio of the [Ca^2+^]_i_ peak heights (second over first peak) of individual cells. Data are presented as mean ± SEM; n.s., not significant (*n* = 90 cells for Tyrode, *n* = 60 cells for DEA/NO, measured on twelve coverslips obtained from two independent cultures).

To evaluate a potential crosstalk of NO-cGMP and Ca^2+^ signaling, CGNs from R26-CAG-cGi500(L1) mice were loaded with the fluorescent Ca^2+^ indicator Fura-2, and then cGMP and Ca^2+^ were monitored simultaneously (Figure 2). Experiments were conducted under continuous flow to allow for the addition of different substances to investigate their influence on cGMP and Ca^2+^ signaling in real time. As shown in Figure 2A, brief repetitive stimulation of CGNs with glutamate (100 μM) induced reliable Ca^2+^ transients (black trace), but no changes of the intracellular cGMP concentration (green trace). On the other hand, DEA/NO (100 nM) increased the cGMP level but had no effect on the intracellular Ca^2+^ concentration ([Ca^2+^]_i_) under baseline conditions (Figure 2B). However, when glutamate was applied in the presence of DEA/NO and an elevated cGMP level, glutamate-induced Ca^2+^ transients were significantly potentiated when compared to control Ca^2+^ transients in the absence of DEA/NO (Figure 2A–C). To evaluate the effect of NO-induced cGMP on the glutamate-induced rise in the Ca^2+^ concentration, the peak height of the second Ca^2+^ transient (in the presence of Tyrode buffer or DEA/NO) was divided by the peak height of the first Ca^2+^ transient (in the absence of DEA/NO). Stimulation of CGNs with glutamate during superfusion with DEA/NO resulted in a significantly augmented [Ca^2+^]_i_ compared to control cells that were stimulated with glutamate and superfused with Tyrode buffer, with a second peak to first peak height ratio of ≈1.2 versus ≈0.7 (Figure 2C; *p* < 0.001). These data depicted a NO-induced augmentation of glutamate-induced Ca^2+^ transients in primary mouse CGNs.

NO could have influenced [Ca^2+^]_i_ via a cGMP-dependent or -independent mechanism. To determine the importance of cGMP in the observed augmented [Ca^2+^]_i_, CGN cultures were prepared from cGMP sensor mice, which lacked the β_1_ subunit of NO-GC (NO-GC KO) [30] and, therefore, expressed no functional NO-GC. These CGNs showed normal glutamate-induced Ca^2+^ transients (Figure 2D, black trace, and Figure 2F, Tyrode). As expected, NO-GC KO CGNs showed no increase in the cGMP concentration upon addition of DEA/NO (100 nM) (Figure 2E, green trace), confirming the absence of functional NO-GC in these cells. In contrast to wild-type CGNs (Figure 2A–C), NO-GC KO cells showed similar glutamate-induced Ca^2+^ transients in the absence and presence of DEA/NO (Figure 2D–F). These results strongly supported the notion that NO potentiation of [Ca^2+^]_i_ in CGNs is mediated by activation of NO-GC and generation of cGMP.

### 2.3. Use of cGMP Analogues Confirms cGMP-Dependent Increase of Glutamate-Induced Ca^2+^ Transients

**Figure 3:**
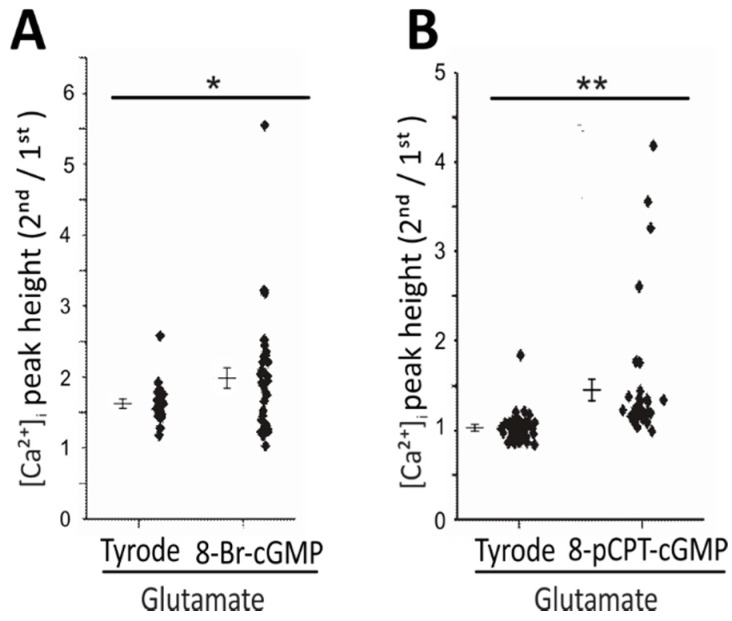
The addition of cGMP analogues confirmed cGMP–Ca^2+^ crosstalk in CGNs. (**A**) Quantification of glutamate (100 µM, 10 s)-induced elevation of [Ca^2+^]_i_ in the absence of cGMP analogue (Tyrode) or after pre-incubation of cells with 100 µM 8-Br-cGMP for 5.5 min. Data are depicted as the ratio of the [Ca^2+^]_i_ peak heights of individual cells (second over first peak, where the second peak was induced by glutamate in the presence of Tyrode or 8-Br-cGMP) and presented as mean ± SEM; * *p* < 0.05 (*n* = 15 cells for Tyrode, *n* = 34 cells for 8-Br-cGMP, measured on seven coverslips obtained from three independent cultures). (**B**) Quantification of glutamate (100 µM, 10 s)-induced elevation of [Ca^2+^]_i_ in the absence of cGMP analogue (Tyrode) or after pre-incubation of cells with 100 µM 8-pCPT-cGMP for 5.5 min. Data are depicted as the ratio of the [Ca^2+^]_i_ peak heights of individual cells (second over first peak, where the second peak was induced by glutamate in the presence of Tyrode or 8-pCPT-cGMP) and presented as mean ± SEM; ** *p* < 0.01 (*n* = 29 cells for Tyrode, *n* = 22 cells for 8-pCPT-cGMP, measured on six coverslips obtained from two independent cultures).

To further test the importance of cGMP in augmented glutamate-induced Ca^2+^ signals, CGNs were stimulated with glutamate (100 μM) during superfusion with the membrane-permeable cGMP analogue 8-Br-cGMP (100 μM) or 8-pCPT-cGMP (100 μM). In the presence of 8-Br-cGMP or 8-pCPT-cGMP, glutamate-induced Ca^2+^ transients were significantly augmented compared to controls without cGMP analogues (Figure 3A, *p* < 0.05; Figure 3B, *p* < 0.01). In the experiments with 8-Br-cGMP, we noted that the peak height ratio of the second over the first Ca^2+^ transient under control conditions (Tyrode) was ≈1.6 (Figure 3A). This value was higher than the control ratios obtained in the other experiments, which were ≈0.8–1.0 (Figure 2C,F and Figure 3B). The variability of control transients might be related to different growth states of different CGN cultures. However, it is important to note that control cells (treated with Tyrode) and experimental cells (treated with DEA/NO or cGMP analogues) were always measured side-by-side with the same batch of cells. Therefore, the potential variability in the control ratio between different CGN cultures should not have been a major confounding factor in these experiments. The results with cGMP analogues further supported a cGMP–Ca^2+^ crosstalk in CGNs, which could be mediated via cGKs, CNG channels, or other cGMP effector proteins.

### 2.4. Western Blot Analysis Does Not Detect cGKI or cGKII Expression in CGNs

**Figure 4:**
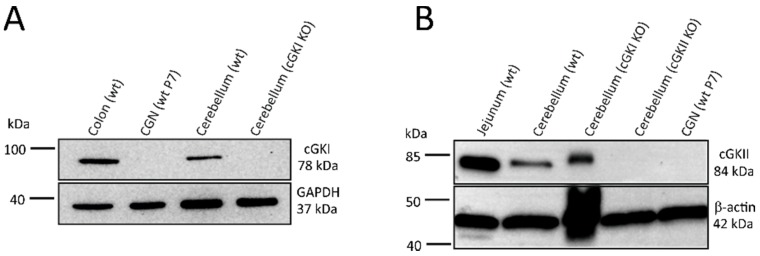
Western blot analysis failed to detect cGKI or cGKII expression in primary CGNs. (**A**) Lysates prepared from CGN cultures grown for 5 days in vitro (10 μg), colon (10 μg), and cerebellum of a wild type mouse (wt) (10 μg), as well as cerebellum of a cGKI knockout mouse (cGKI KO) (10 μg) were stained with a highly specific cGKI antibody. GAPDH was used as a loading control. Representative data from three independent CGN primary cultures are shown. (**B**) Lysates prepared from CGN cultures grown for 5 days in vitro (30 μg), jejunum (20 µg), and cerebellum of a wild type mouse (wt) (70 µg), as well as cerebellum of a cGKI knockout mouse (cGKI KO) (70 µg) and of a cGKII knockout mouse (cGKII KO) (70 µg) were stained with a highly specific cGKII antibody. β-actin was used as a loading control. Representative data from two independent CGN primary cultures are shown. Expected molecular weights of respective proteins are indicated on the right. CGN cultures were established from 7-day-old wild type mice (wt P7) and extracts of the cerebellum and internal organs were prepared from 8- to 25-week-old mice.

To determine if cGKs could be downstream targets of cGMP, lysates from our CGN primary cultures were analyzed via Western blot with highly specific cGKI and cGKII antibodies (Figure 4). Both antibodies were validated with respective knockout tissues to confirm their specificity. While the cGKI protein was readily detected in lysates from colon and cerebellum isolated from wild type mice, it was not detected in primary CGNs (Figure 4A). The cGKII protein was detected in lysates from jejunum of wild type mice, as well as in cerebellum isolated from wild type or cGKI knockout mice, but it was not detected in the our CGN lysates (Figure 4B).

### 2.5. Real-Time cGMP Imaging in CGNs Reveals cGMP Degradation via Zaprinast-Sensitive PDEs

**Figure 5:**
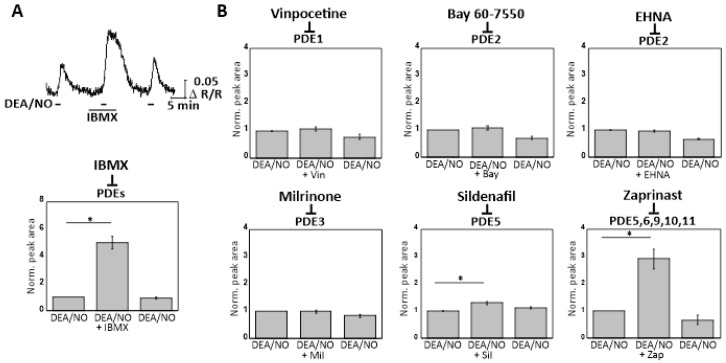
Real-time cGMP imaging in CGNs reveals degradation of cGMP by Zaprinast-sensitive PDEs. (**A**) cGi500-expressing CGNs isolated from R26-CAG-cGi500(L1) mice were stimulated three times with DEA/NO (50 nM), first with DEA/NO alone, then in the presence of IBMX (100 µM), and finally again with DEA/NO alone. The bar graph shows the statistical evaluation of DEA/NO-induced cGMP peak areas before (first peak), during (second peak), and after (third peak) incubation with IBMX. Peak areas were normalized to the first peak of each experiment. Data are presented as mean ± SEM; * *p* < 0.05 first versus second peak (*n* = 6 cells measured on three coverslips obtained from three CGN cultures). (**B**) As described for (**A**), CGNs were superfused with DEA/NO (50 nM) in the absence and presence of indicated PDE inhibitors. The following PDE inhibitors were tested: 5 µM Vinpocetine (Vin); 10 nM Bay 60-7550 (Bay); 10 µM EHNA; 10 µM Milrinone (Mil); 20 µM Sildenafil (Sil); 20 µM Zaprinast (Zap). Data are presented as mean ± SEM; * *p* < 0.05 first versus second peak (*n* = 6–12 cells per condition; cells were measured on two coverslips obtained from two CGN cultures).

To evaluate which PDE(s) degrade cGMP in murine CGNs, the cells were exposed to various PDE inhibitors. CGNs from cGMP sensor mice were first superfused with DEA/NO (50 nM) alone (Figure 5A, first peak), and then with the general PDE inhibitor IBMX (100 µM) followed by DEA/NO (50 nM) in the presence of IBMX (Figure 5A, second peak) and a final application of DEA/NO (50 nM) alone (Figure 5A, third peak). IBMX alone had no influence on the basal cGMP level in cultured CGNs, however, it strongly potentiated the cGMP increase elicited by DEA/NO compared to DEA/NO alone (Figure 5A). To identify the PDEs that play a role in cGMP degradation in CGNs, specific PDE inhibitors [13] were applied in the same manner as shown for IBMX in Figure 5A. These PDE inhibitors were Vinpocetine (5 µM) for PDE1, Bay 60-7550 (10 nM) for PDE2, EHNA (10 µM) for PDE2, Milrinone (10 µM) for PDE3, Sildenafil (20 µM) for PDE5, and Zaprinast (20 µM) for PDE5, 6, 9, 10, and 11. These measurements (Figure 5B) revealed a weak but significant effect of Sildenafil (*p* < 0.05) and a strong effect of Zaprinast (*p* < 0.05). The other tested PDE inhibitors did not show statistically significant effects on NO-induced cGMP elevation. These data implicated PDE5, 6, 9, 10, and 11 as possible cGMP-degrading PDEs in murine CGNs.

## 3. Discussion

In this study, we have investigated NO-cGMP signal transduction in CGNs by real-time imaging of cGMP and Ca^2+^. cGMP imaging in acute cerebellar slices and individual CGNs in primary culture revealed that NO, but not ANP and CNP, was able to induce an increase in cGMP, which indicated that the cGMP synthesizing enzyme NO-GC was present, while particulate guanylyl cyclases stimulated by ANP or CNP were not. Importantly, we have identified a NO-cGMP-dependent augmentation of glutamate-induced Ca^2+^ transients in CGNs. These results complement other research that has demonstrated NO-cGMP signaling in CGNs [21,31]. A recent report used cGMP imaging in acute brain slices of cGMP sensor mice to demonstrate NO-induced cGMP elevations in the cerebellum, striatum, and hippocampus [32]. In contrast to the present study, which was conducted with transgenic mice expressing the cGMP sensor ubiquitously, Peters and colleagues used a neuron-specific cGMP sensor mouse model. In this model, they also found robust NO-induced cGMP signals in the granule cell layer of the cerebellum [32], thus, providing further support for the presence of a NO-cGMP signalling cascade in CGNs in vivo.

With the knowledge that NO was able to induce a robust cGMP response in CGNs, we investigated a potential crosstalk between the NO-cGMP pathway and glutamate-induced Ca^2+^ signaling via simultaneous imaging of cGMP and Ca^2+^ in individual CGNs in primary cultures. Brief repetitive stimulation of our CGNs with glutamate evoked robust Ca^2+^ transients. The glutamate-induced Ca^2+^ elevations were likely mediated by the NMDA receptor [33], but we could not exclude other types of glutamate receptors as sources of Ca^2+^ entry. It has been reported that upon activation of Ca^2+^-permeable NMDA-type glutamate receptors in the brain, NO synthase is activated and NO synthesized, which then activates NO-GC and elevates intracellular cGMP levels [25,26]. Other work has proposed that Ca^2+^ entry might decrease NO donor-induced cGMP levels in CGNs via activation of Ca^2+^/calmodulin-stimulated PDE1 [34]. However, at no time during the addition of glutamate, in the absence or presence of the NO donor DEA/NO, did we observe a glutamate-/Ca^2+^ entry-triggered change of the cGMP concentration in our CGNs (Figure 2A,B). This could be a result of the short duration that CGNs were exposed to glutamate (10 s), compared to experiments where NMDA receptors were stimulated for minutes [25,26]. Moreover, we did not detect functional PDE1 in our CGNs (Figure 5B).

Interestingly, during exposure of CGNs to DEA/NO, a significant augmentation of glutamate-induced Ca^2+^ transients was observed indicating that NO potentiates Ca^2+^ entry. Given that NO can also produce cGMP-independent effects [1], it was important to determine if the observed NO-induced [Ca^2+^]_i_ augmentation was indeed cGMP-dependent. To investigate this, we analyzed CGNs from knockout mice lacking functional NO-GC. In line with our hypothesis, exposure of NO-GC KO CGNs to NO did not produce cGMP and did not augment glutamate-induced Ca^2+^ transients. To provide further evidence for the importance of cGMP in the potentiation of glutamate-induced elevation of [Ca^2+^]_i_, we exposed CGNs to the membrane-permeable cGMP analogues 8-Br-cGMP and 8-pCPT-cGMP. Indeed, both cGMP analogues were effective and enhanced glutamate-induced elevation of [Ca^2+^]_i_. These results support the notion that NO potentiates glutamate-induced Ca^2+^ transients in murine CGNs via activation of NO-GC and synthesis of cGMP. Interestingly, a recent study reported that the slow alcohol-induced increase of [Ca^2+^]_i_ in CGNs was suppressed by pre-incubation of the cells with 8-Br-cGMP [35]. Thus, it appears that activation of cGMP signaling in CGNs can both increase and decrease [Ca^2+^]_i_ depending on the type of Ca^2+^ signal.

What could be the mechanism downstream of cGMP that augments glutamate-induced Ca^2+^ transients in CGNs? Although 8-Br-cGMP and 8-pCPT-cGMP are commonly used to activate cGKs, it is important to note that these cGMP analogues also bind to and modulate the activity of other cGMP downstream targets such as PDEs and CNG channels [36,37]. Indeed, previous work has reported a slow increase in resting [Ca^2+^]_i_ in Fura-2 loaded rat CGNs upon the addition of 8-Br-cGMP, suggesting the presence of functional CNG channels [38]. However, under our experimental conditions, resting [Ca^2+^]_i_ was not affected by NO-induced endogenous cGMP (Figure 2B) or two cGMP analogues, 8-Br-cGMP and 8-pCPT-cGMP (data not shown). Thus, it is unlikely that the augmentation of glutamate-induced Ca^2+^ transients by these agents, as observed in our study, was related to the activation of CNG channels. Rat CGNs also express hyperpolarization-activated cyclic nucleotide-gated channels [39]. However, because these ion channels are principally activated by cAMP and not cGMP, it is unlikely that they were directly involved in the effects of our test compounds. Future studies are required to identify the cGMP effector mechanism behind the potentiation of glutamate-induced Ca^2+^ transients in CGNs by NO-cGMP signaling.

Previous work with genetically-modified mice has demonstrated important roles of cGKI in the brain, from modulation of synaptic plasticity in hippocampal neurons [40] and cerebellar Purkinje cells [20] to the regulation of sleep-wake activity [41]. Interestingly, cGKI also mediates cGMP–Ca^2+^ crosstalk in vascular smooth muscle cells. However, activation of cGKI in these cells typically results in suppression of agonist-evoked increases of [Ca^2+^]_i_ [42,43,44], suggesting that cGKI is not the effector of the NO-cGMP-mediated increase of [Ca^2+^]_i_ observed in CGNs. Indeed, Western blot analysis did not detect cGKI or cGKII protein expression in our primary mouse CGNs. These findings contrast with previous studies of the Torres lab. These authors detected cGKI mRNA and protein and cGKII mRNA in CGNs via qPCR and Western blotting, and they reported important roles of cGKI and cGKII in synaptic vesicle recycling in CGNs [21,31,45,46]. The discrepancy in expression data could be explained by methodological differences. First, we did not analyze mRNA levels but used highly specific antibodies, which we validated with knockout tissues, to detect cGKI and cGKII proteins. Torres and colleagues used a different cGKI antibody with presumably different specificity and sensitivity for Western blotting, and they did not analyze cGKII expression at the protein level. It is interesting to note that while we were not able to detect cGKI and cGKII in our CGN lysates, both proteins were readily detected in extracts of whole cerebellum. This suggests that cell types other than CGNs express cGKI and/or cGKII in the cerebellum. Indeed, immunohistochemical staining of cerebellar sections showed strong expression of cGKI in cerebral vessels and Purkinje neurons, but no evidence for its expression in CGNs [10,20]. Another reason for discrepant results regarding the expression and functional relevance of cGKs and CNG channels could be that CGNs develop a differential expression of these cGMP pathway components upon culturing over time [21,38]. Moreover, most previous studies of NO-cGMP signaling in CGNs were done in rat cells, while we used CGNs from mice. Thus, differences in antibody selectivity and sensitivity, culture conditions/times, as well as species differences might account for differences in protein expression observed in our studies and those by Torres and co-workers. Our studies, as well as those by Torres et al., were performed with CGNs isolated from young (P7) mice or rats. Since cGMP signaling in the brain may dramatically change with aging [47], we should be careful with extrapolating our findings to CGNs in the adult cerebellum.

cGMP degradation occurs via PDEs and many of the eleven PDE families have been reported in the CNS [13,48,49]. To our knowledge, the PDEs involved in cGMP degradation in CGNs have not been systematically characterized. With our FRET-based cGi500 sensor, it was possible to investigate the role of PDEs in cGMP degradation in individual living CGNs in real time (Figure 5B). NO-induced cGMP levels were not affected by inhibitors of PDE1, 2, and 3, while they were strongly potentiated by Zaprinast, which inhibits PDE5, 6, 9, 10, and 11. Addition of the PDE5 inhibitor Sildenafil resulted in a small increase in cGMP (Figure 5B). Our findings are consistent with the reported expression of PDE9 in rat CGNs [50] and of PDE9, but not PDE2, in CGNs of the human brain [51]. Previous research performed in rodents and humans has suggested that PDE9, 10, and 11 play a role in neurological diseases, such as PDE9 in bipolar affective disorder [52], PDE10 in social interaction, schizophrenia, and bipolar disorder [53,54,55], and PDE11 in major depression [56]. However, neither PDE6 nor PDE11 are expressed in the cerebellum [13,49,57]. PDE9 appears to be localized to the membrane and nuclear fractions of cerebellar tissue [58]. In heart muscle, PDE9 specifically regulates cGMP pools that are generated by membrane-associated particulate guanylyl cyclases rather than cytosolic NO-GC [59]. Thus, we propose that PDE5 and PDE10 are the most likely candidates that degrade NO-induced cGMP in murine CGNs.

Several imaging-based studies have identified important functions of cGMP, cAMP, and Ca^2+^ in neuronal cells and, interestingly, complex relationships between the spatiotemporal dynamics of these signaling molecules. For instance, reciprocal regulation of cAMP and cGMP modulates axon/dendrite formation in hippocampal neurons [60]. In this study, FRET imaging showed that alterations of the amount of cAMP resulted in opposite changes in the amount of cGMP, and vice versa, through the activation of PDEs and protein kinases. Another study showed that the NO-cGMP pathway inhibits transient cAMP signals through the activation of PDE2 in striatal neurons [15]. Recently, an interplay among cGMP, cAMP, and Ca^2+^ has been described in olfactory sensory neurons [61] and dorsal root ganglion neurons [62,63], as well as an association of this signaling crosstalk with axonal pathfinding and neurotransmitter release. Thus, it is tempting to speculate that the cGMP–Ca^2+^ crosstalk in CGNs identified in the present study also involves cAMP. In the future, it will be interesting to monitor cGMP/cAMP and cAMP/Ca^2+^ simultaneously in individual CGNs with the use of spectrally compatible fluorescent indicators. With these experiments, one could test the hypothesis that NO-cGMP signaling modulates the cAMP level, presumably via regulation of cAMP-degrading PDEs, and that cAMP in turn regulates glutamate-induced Ca^2+^ transients in CGNs.

In sum, real-time imaging of cGMP and Ca^2+^ combined with expression analysis of cGKI and cGKII indicates the presence of a cGK-independent NO-cGMP signaling cascade that potentiates glutamate-induced Ca^2+^ transients in murine CGNs, and a Zaprinast-sensitive degradation of cGMP in these cells. These findings deepen our mechanistic understanding of cGMP signaling in the CNS. The cGMP–Ca^2+^ crosstalk reported here likely influences neurotransmitter-stimulated functions of CGNs and perhaps other neuronal cell types and might be targeted by cGMP-modulating medications to treat neurological disorders.

## 4. Materials and Methods

### 4.1. Mice

All animal procedures were performed in compliance with the humane care and use of laboratory animals and approved by the local authority (Regierungspräsidium Tübingen, IB 1/15, 21 August 2015). Mice were housed at 22 °C and 50–60% humidity in a 12 h light/12 h dark cycle with access to standard rodent chow and tap water. The following mouse lines were used: R26-CAG-cGi500(L1) [27], NO-GC KO [30], and R26-CAG-cGi500(L1);NO-GC KO cross mice. Genotyping of the animals was performed by PCR analysis of ear puncture DNA using the following primers: for detection of the R26-CAG-cGi500(L1) allele, BB01 (CTCTGCTGCCTCCTGGCTTCT), BB02 (CGAGGCGGATCACAAGCAATA) and BB03 (TCAATGGGCGGGGGTCGTT), which amplify a 330-bp fragment of the wild type allele (BB01 and BB02) and a 250-bp fragment of the transgene (BB01 and BB03); for NO-GC KO mice, BB19 (AAGATGCTGAAGGGAAGGATGC), BB20 (CAGCCCAAAGAAACAAGAAGAAAG) and BB21 (GATGTGGGATTGTTTCTGAGGA), which amplify a 680-bp fragment of the wild type allele (BB19 and BB20) and a 830-bp fragment of the knockout allele (BB19 and BB21).

### 4.2. Drugs

The following drugs were used: ANP and CNP (Tocris, Bristol, UK), DEA/NO (Axxora, Ann Arbor, MI, USA), glutamate (Sigma, St. Louis, MO, USA), IBMX (Sigma), Vinpocetine (Cayman, Ann Arbor, MI, USA), Bay 60-7550 (Cayman), EHNA (Axxora), Milrinone (Sigma), Sildenafil (Cayman), Zaprinast (Santa Cruz, Dallas, TX, USA), 8-Br-cGMP (Biolog, Bremen, Germany), and 8-pCPT-cGMP (Biolog).

### 4.3. Cell Culture

To establish primary cultures of cerebellar granule neurons (CGNs) from mice, the following solutions and “tubes” were prepared: 10× Krebs buffer (1.24 M NaCl, 54 mM KCl, 5 mM NaH_2_PO_4_, pH 7.4); 0.3% bovine serum albumin (BSA, Roth, Karsruhe, Germany) solution in 1× Krebs buffer, 14.3 mM d-glucose, 2.5 mM MgSO_4_, sterilized by filtration; “tube 1” contained 30 mL 0.3% BSA solution; “tube 2” contained 30 mL 0.3% BSA solution and 300 μL trypsin solution (2.5× trypsin-EDTA (Thermo Scientific, Waltham, MA, USA) in PBS), added shortly before use; “tube 3” contained 7.8 mg trypsin inhibitor (Life Technologies, Carlsbad, CA, USA) in 15 mL 0.3% BSA solution, 3.1 mM MgSO_4_, and 0.1 mg/mL DNase (Roche, Basel, Switzerland), added shortly before use); in “tube 4”, 17 mL 0.3% BSA solution weas mixed with 8 mL solution from “tube 3” and then 10 mL was discarded, so that 15 mL remained in “tube 4”; “tube 5” contained 12.5 mL 0.3% BSA solution, 2.5 mM MgSO_4_, and 0.1 mM CaCl_2_. Two to five cerebella from 7-day-old mice were isolated and the surrounding meninges were removed in 0.3% BSA solution. Then, the cerebella were homogenized in 2 mL 0.3% BSA solution with a pipette, transferred into “tube 1” and centrifuged at 170× *g* for 5 min at room temperature. The supernatant was removed, and the pellet was resuspended in the solution from “tube 2” and incubated for 15 min at 37 °C with gentle shaking every 5 min. Then, the suspension was transferred into “tube 4”, mixed, and centrifuged at 170× *g* for 5 min at room temperature. The supernatant was removed, and the cell pellet was resuspended in the solution from “tube 3” by pipetting 10 times with a Pasteur pipette. Then, the suspension was transferred into “tube 5”, mixed, passed through a netwell mesh (70 µm), and centrifuged at 170× *g* for 5 min at room temperature. The pellet was resuspended in CGN medium (Minimal Essential Medium (Life Technologies) containing 22 mM KCl, 2% B27 supplement (Thermo Scientific), 9% fetal bovine serum (Thermo Scientific), 0.3 mM glutamine (Thermo Scientific), and 0.1 mM gentamicin (Thermo Scientific)) and plated on 24-well plates (100 k cells per well, viability > 95% as determined by trypan blue staining). Wells were equipped with glass coverslips, which had been coated overnight with poly-d-lysine (20 µg/mL, Thermo Scientific). CGNs were grown at 37 °C and 5% CO_2_, and after 24 h cytosine arabinoside (5 µM, Sigma) was added. Every 3 days, half of the medium was changed.

### 4.4. Imaging

FRET/cGMP and Ca^2+^ imaging of cultured CGNs was performed 5–7 days after plating using an epifluorescence setup as described previously [27,29,64]. Briefly, the setup consisted of an inverted Axiovert 200 microscope (Zeiss, Oberkochen, Germany) equipped with a NeoFluar40x/1.30 oil objective, a light source with excitation filter switching device (Oligochrome, TILL Photonics GmbH, Graefelfing, Germany), a Dual-View beam splitter (Photometrics) with 516 nm dichroic mirror and emission filters for CFP (480/30 nm) and YFP (535/40 nm), and a charge-coupled device camera (Retiga 2000R; QImaging, Surrey, BC, Canada).

cGMP imaging was performed with CGNs from R26-CAG-cGi500(L1) mice expressing the cGi500 sensor. For simultaneous imaging of cGMP and Ca^2+^, coverslips with cGMP sensor cells were incubated with 2.5 µM Fura-2AM (Calbiochem, San Diego, CA, USA; 1 mM stock solution in DMSO) in Tyrode buffer (140 mM NaCl, 5 mM KCl, 1.2 mM MgSO4, 2.5 mM CaCl2, 5 mM d-glucose, 5 mM HEPES, pH 7.4) for 35 min at 37 °C in the dark before measurement. Coverslips were mounted into a Warner Instrument SA-20LZ superfusion imaging chamber (Harvard Bioscience, Holliston, MA, USA) and superfused at 37 °C at a flow rate of 1 mL/min with Tyrode buffer or Tyrode buffer supplemented with drugs. Test compounds were applied via Pharmacia IV-7 injection valves (GE Healthcare, Chicago, IL, USA) with either 7.0, 2.0, or 0.16 mL sample loops. Cells were imaged at 40× magnification and 4 × 4 binning. Signals were recorded at a frequency of 5 Hz or 1 Hz for cGMP imaging alone or simultaneous cGMP/Ca^2+^ imaging, respectively. For Fura-2-based Ca^2+^-imaging, fluorescence was excited at 340/26 nm and 387/11 nm (15% lamp intensity each) and emission was recorded in the 535/40 nm channel (65 ms and 20 ms exposure time with the respective excitation wavelength). For cGMP imaging with cGi500, excitation was performed at 445/20 nm (45% lamp intensity) and emission recorded simultaneously at 480/30 nm and 535/40 nm (40 ms exposure).

Acute brain slices were prepared with a vibratome and imaged with a spinning disk microscope essentially as described in an accompanying study [32]. The setup consisted of an upright Examiner.Z1 microscope (Zeiss, Oberkochen, Germany), a Yokogawa CSU-X1 spinning disk confocal scanner, three diode lasers (445 nm, 488 nm and 561 nm), three water immersion objectives (W N-ACHROMAT 10x/0.3, W Plan-APOCHROMAT 20x/1.0 DIC (UV) VIS-IR, W Plan-APOCHROMAT 40x/1.0 DIC VIS-IR; all from Zeiss) and one air objective (EC Plan-NEOFLUAR 2.5x/0.085; Zeiss). For FRET-based cGMP imaging, the donor fluorophore CFP was excited with the 445 nm laser, and a Dual-View beam splitter (Photometrics) with 505 nm dichroic mirror, and 470/24 nm and 535/30 nm emission filters was used for simultaneous acquisition of CFP and YFP. Signals were recorded with an electron-multiplying charged-coupled device (EM-CCD) camera (QuantEM 512SC, Photometrics) at a frame rate of 0.2 Hz and an exposure time of 300 ms. The system was controlled by VisiView software (Visitron Systems). A CoolLED pE-2 LED system was used for epifluorescence illumination at 400 nm, 450 nm, 500 nm, and 561 nm. During real-time imaging, the tissue was continuously superfused with carbogen-gassed Ringer buffer (126 mM NaCl, 2.5 mM KCl, 1 mM MgCl_2_, 1 mM CaCl_2_, 1.25 mM NaH_2_PO_4_, 26 mM NaHCO_3_, 20 mM d-glucose) or Ringer buffer containing drugs of interest at a flow rate of 1 mL/min at 37 °C. A custom-built superfusion system was used consisting of a FPLC pump (Pharmacia P-500, GE Healthcare), FPLC injection valves (Pharmacia V-7, GE Healthcare), a magnetic platform (Warner instruments, Holliston, MA, USA), a superfusion chamber (RC-26, Warner Instruments), a Slice Hold-Down (SHD-26H/10, Warner Instruments), and a sample loop (2 mL). To remove the buffer from the system, a vacuum pump with adjustable vacuum (Laboport N86, KNF Neuberger, Freiburg im Breisgau, Germany) was connected to the system [27,64].

### 4.5. PDE Inhibitor Experiments

cGMP measurements with various PDE inhibitors were performed on the same microscopy setup as described in 4.4. Primary CGNs were first stimulated for 2 min with DEA/NO (50 nM) alone and then superfused for 5 min with PDE inhibitor followed by 2 min with DEA/NO (50 nM) and PDE inhibitor in combination, and a final 2-min stimulation with DEA/NO (50 nM) alone. Subsequent drug applications were performed after the preceding FRET signal had returned to baseline.

### 4.6. Protein Isolation and Western Blotting

CGNs on coverslips were treated with lysis buffer (0.67% SDS (*w*/*v*), 21 mM Tris/HCl (pH 8.3), 0.2 mM phenylmethylsulfonylfluoride, and one tablet of PhosSTOP™ phosphatase inhibitor cocktail (Roche) per 10 mL). Protein concentrations of the cell lysates were measured with the Total Protein Kit, Micro Lowry, Peterson’s modification (Sigma). Cell lysates were subjected to SDS-PAGE and Western blot analysis on polyvinylidenefluoride membranes. Membranes were stained with the following antibodies: rabbit polyclonal anti-cGKI (1:5000, [65]), rabbit monoclonal anti-GAPDH (1:1000, Cell Signaling, Danvers, MA, USA, 2118), rabbit polyclonal anti-cGKII (1:100, [66]), or rabbit polyclonal anti-β-actin (1:2000, Abcam, Cambridge, UK, 8227). Antibody binding was detected using HRP-conjugated secondary antibodies (goat anti-rabbit IgG, 1:2000, Cell Signaling, 7074; or 1:7500, Dianova, 111-035-003) and a chemiluminescent substrate (Advansta WesternBright ECL, Biozym; or ClarityTM Western ECL Substrate, Bio-Rad Laboratories, Hercules, CA, USA). Signals were recorded with a CCD camera (Alpha-Imager, Bio-Rad Laboratories; or ChemiDoc system, Bio-Rad Laboratories). Further analysis was performed with Fiji (NIH, Bethesda, MD, USA) [67] or ImageLab software (Bio-Rad Laboratories). Tissues were obtained from 8- to 25-week-old wild-type mice on a C57BL/6 or mixed C57BL/6 × 129/Sv genetic background. Tissues were dissected in PBS and then homogenized in lysis buffer using a FastPrep homogenizer (MP Biomedicals, Santa Ana, CA, USA) or ultra-turrax (IKA, Staufen, Germany).

### 4.7. Immunostaining of Cells

CGNs on coverslips were washed two times with PBS and then treated with ice-cold immuno-fix (3.7% formaldehyde in PBS) for 10 min. The cells were then washed three times with 0.5% BSA (in PBS) and incubated in 5% Normal Goat Serum (in 0.5% BSA solution) for 1 h at room temperature. The cells were then washed two times with 0.5% BSA and incubated with mouse monoclonal antibody against βIII-tubulin (Cell Signaling, 4466, 1:1000 in 0.5% BSA with 0.01% Triton X-100) for 1 h at room temperature. The cells were then washed two times with 0.5% BSA and then incubated with secondary antibody conjugated with Alexa 488 (goat anti-mouse IgG, Cell Signaling, A-11029, 1:100 in 0.5% BSA with 0.01% Triton X-100) and 1 µg/mL Hoechst 33258 (Sigma) for 30 min in the dark at room temperature. The cells were then washed three times with 0.5% BSA and photographed with the microscope setup described in Section 4.4.

### 4.8. Data Analysis and Statistics

Imaging data were analysed as previously described [27]. For image acquisition and online analysis, VisiView (Visitron, Puchheim, Germany) was used, and for offline analysis Fiji software (NIH) [67]. For further analysis, Microsoft Excel (Microsoft Corp., Redmond, WA, USA) and Origin (OriginLab Corp., Northampton, MA, USA) were used. F_480_ signals (CFP emission at 480 nm, cyan traces in respective graphs) and F_535_ signals (YFP emission at 535 nm, orange traces in respective graphs) were background-corrected and used to calculate the F_480_/F_535_ ratio R (green traces in respective graphs). F_340_ signals (intensity of emission at 535 nm after excitation at 340 nm) and F_387_ signals (intensity of emission at 535 nm after excitation at 387 nm) were background-corrected and used to calculate the F_340_/F_387_ ratio R (black traces in respective graphs). ΔF_480_/F_480_, ΔF_535_/F_535_, ΔF_340_/F_340_, ΔF_387_/F_387_ and corresponding ΔR/R traces were obtained by normalization to the baseline recorded for ≈3 min at the beginning of each experiment. For ΔR/R peak area and peak height calculation, Peak Area/Height Analyzer of Origin was used. Peak borders were defined manually. Statistical analysis was performed using Origin software. Statistical differences between more than two groups were analyzed by one-way ANOVA followed by Bonferroni’s test. *p* values < 0.05 were considered to be significant.

## Abbreviations

ANP	Atrial natriuretic peptide
BSA	Bovine serum albumin
[Ca^2+^]_i_	Intracellular Ca^2+^ concentration
CAG	CMV immediate early enhancer, chicken β-actin, and rabbit β-globin
CFP	Cyan fluorescent protein
cGi	cGMP indicator
cGK	cGMP-dependent protein kinase
cGMP	Cyclic 3′ 5′ guanosine monophosphate
CGN	Cerebellar granule neuron
CNG	Cyclic nucleotide gated
CNP	C-type natriuretic peptide
CNS	Central nervous system
DEA/NO	2-(*N*,*N*-diethylamino)-diazenolate-2-oxide diethylammonium salt
FRET	Förster/Fluorescence resonance energy transfer
IBMX	3-Isobutyl-1-methylxanthin
NMDA	*N*-methyl-d-aspartate
NO	Nitric oxide
NO-GC	NO-sensitive guanylyl cyclase
NO-GC KO	NO-sensitive guanylyl cyclase knockout
PBS	Phosphate buffered saline
PDE	Phosphodiesterase
YFP	Yellow fluorescent protein
